# Coacervate Droplets as Biomimetic Models for Designing Cell‐Like Microreactors

**DOI:** 10.1002/marc.202400626

**Published:** 2024-11-26

**Authors:** Tsvetomir Ivanov, Thao. P. Doan‐Nguyen, Mohammed Amin Belahouane, Zhen Dai, Shoupeng Cao, Katharina Landfester, Lucas Caire da Silva

**Affiliations:** ^1^ Department of Physical Chemistry of Polymers Max Planck Institute for Polymer Research Ackermannweg 10 55128 Mainz Germany; ^2^ Department of Chemistry McGill University H3A 0B8 Montreal Canada; ^3^ College of Polymer Science and Engineering Sichuan University Chengdu 610065 P. R. China

**Keywords:** bio‐mimicking, catalysis, coacervates, compartments, life‐like materials

## Abstract

Coacervates are versatile compartments formed by liquid–liquid phase separation. Their dynamic behavior and molecularly crowded microenvironment make them ideal materials for creating cell‐like systems such as synthetic cells and microreactors. Recently, combinations of synthetic and natural molecules have been exploited via simple or complex coacervation to create compartments that can be used to build hierarchical chemical systems with life‐like properties. This review highlights recent advances in the design of coacervate compartments and their application as biomimetic compartments for the design of cell‐like chemical reactors and cell mimicking systems. It first explores the variety of materials used for coacervation and the influence of their chemical structure on their controlled dynamic behavior. Then, the applications of coacervates as cell‐like systems are reviewed, focusing on how they can be used as cell‐like microreactors through their ability to sequester molecules and provide a distinct and regulatory microenvironment for chemical reactions in aqueous media.

## Graphical abstract

Coacervates are versatile analogs of biomolecular condensates formed by liquid–liquid phase separation. They are adaptable, life‐like compartments with unique chemical environments that allow the design of cell‐like microreactors with enhanced performance and adaptable properties. This review discusses recent advances in the design of coacervate droplets and their application as cell‐like microreactors.

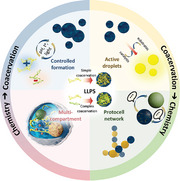

## 1 Introduction

**Scheme 1:**
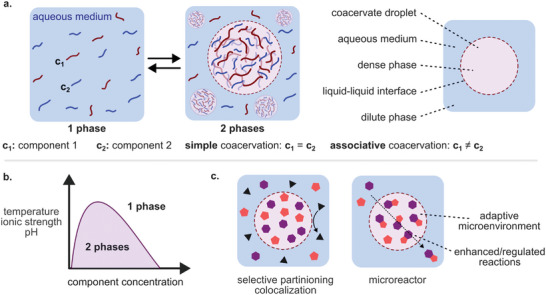
Coacervation formation and coacervate‐based microreactors. a) Coacervate formation via simple and associative coacervation. b) Simplified phase diagram showing the phase separation as a function of component concentration and system variables. c) Illustration of coacervates as microreactors.

Liquid–liquid phase separation (LLPS) is a phenomenon that occurs when a solution containing one or more components spontaneously separates into two or more distinct liquid phases, each containing different concentrations of the components.^[^ ^1^ ^]^ This reversible process results in the spontaneous formation of molecularly crowded micron‐sized droplets characterized by the lack of a membrane and low interfacial tension. Liquid–liquid phase separation can occur through three basic types of interactions: segregative, associative, and simple phase separation between one or more molecules.^[^ ^2^ ^]^ Segregative phase separation occurs between two soluble molecules that do not mix despite a favorable mixing entropy, often due to repulsive interactions between the molecules. A classic example of such a system is the phase separation between polyethylene glycol (PEG) and dextran (Dex) in aqueous solution.^[^ ^3^ ^]^ Associative phase separation is defined by attractive interactions between two or more soluble molecules that end up in the same condensed phase. The condensed coacervate phase is enriched in both solutes but still contains a significant amount of the solvent (typically water), while the dilute phase contains mostly the solvent with a low concentration of the solutes.^[^ ^4^ ^]^ An example of associative phase separation are the coacervates formed via LLPS of oppositely charged polyelectrolytes.^[^ ^5^ ^]^ Finally, simple phase separation is observed when the attractive interaction forces are present in a single molecule, causing it to phase separated under certain physicochemical conditions.^[^ ^6^, ^7^ ^]^ Associative and simple coacervation are illustrated in **Scheme** **1**.

The ability of coacervates to regulate chemistry was first recognized by their function in biological systems: in cells, biomacromolecules such as proteins or nucleic acids can undergo LLPS, resulting in liquid‐like, molecularly crowded droplets.^[^ ^8^ ^]^ This immiscible liquid phase allows selective partitioning and regulated transfer and concentration of molecules, which is exploited by cells to regulate biochemical processes. Examples of regulatory biomolecular condensates in cells include nucleoli, P granules, and stress granules, which are essential for the regulation of biochemical processes in the cell. The importance of biomolecular condensates in biology and the study of coacervates in general has led to insights into prebiotic compartments that may have played a role in the origin of life.^[^ ^9^ ^]^ In these scenarios, coacervate droplets are viewed as adaptive, molecularly crowded liquid containers that provided compartmentalization and regulatory mechanisms for prebiotic chemical reactions that eventually led to life.

The unique combination of adaptive formation, selective partitioning of molecules, and differentiated chemical microenvironments makes coacervates interesting materials for creating controlled compartmentalized environments with tunable properties. Although the composition of coacervates in cells is relatively complex, coacervates can be obtained from much simpler, more accessible, and diverse materials such as polymers, polysaccharides, and short peptides. The wide choice of building blocks allows the production of droplets with desired viscosity, density, wetting, encapsulation efficiency, and selectivity.^[^ ^10^, ^11^, ^12^, ^13^ ^]^ These properties make coacervate droplets particularly suitable for the creation of cell‐like chemical reactors or microcompartmentalized reaction hubs, and also artificial organelles due to their ability to selectively sequester and localize active molecules such as enzymes and synthetic catalysts.^[^ ^6^, ^14^ ^]^ In addition, coacervates can be integrated into other compartments, such as vesicle‐based systems, to form hierarchically organized multicompartments that resemble cellular organization.^[^ ^15^, ^16^, ^17^, ^18^ ^]^

Coacervates are also efficient compartments for the stabilization, transport, and delivery of sensitive molecules such as biomolecules and active drugs, which is of great interest for medical applications.^[^ ^19^, ^20^ ^]^ Finally, coacervate droplets provide a platform to study chemical communication involving synthetic compartments via controlled molecular transport between different droplet populations. As such, they provide the building blocks for the design of communicative networks such as synthetic tissue‐like systems.^[^ ^21^ ^]^

In this review, we aim to provide an up‐to‐date synthesis of advances in liquid–liquid phase separation (LLPS) and coacervate droplet formation, with particular emphasis on their use as an adaptive and versatile compartment for the creation of cell‐like microreactors. This review provides a perspective on the design of microreactors that utilize coacervate microenvironment design to enable and regulate chemical reactions in aqueous media, an area that has attracted intense interest in recent years and is rapidly evolving. We begin by introducing different types of building blocks to create coacervates that can be used as compartments for cell‐like microreactors. We then discuss recent developments in the design of adaptive coacervates that respond to external and internal stimuli. Finally, we discuss examples of coacervates as microreactors and cell‐like chemical systems that benefit from the versatility and tunability of coacervates as microenvironments to support, regulate, and enhance chemical reactions. The combination of controlled microenvironment and adaptability allows microreactors to find applications in chemistry and in synthetic cell design, bridging the gap between materials science, catalysis, drug delivery, and synthetic biology.

## 2 Coacervate Building Blocks

In recent years, a variety of natural and synthetic materials have been developed and used to form coacervate droplets. Some representative examples are shown in **Table** **1**. In cells, biomolecular condensates are used for compartmentalization, consisting mostly of disordered proteins and nucleic acids held together by weak intermolecular forces. Inspired by this, researchers have developed components that can form synthetic coacervates that mimic the behavior and properties of biomolecular condensates, such as dynamic assembly and disassembly, and selective and tunable sequestration of molecules. Examples of common components include polyelectrolytes, polypeptides, nucleic acids, and polysaccharides.

**Table 1:** Types of coacervation based on the chemical nature of the components.

Coacervation type	Materials nature	Components	References
Simple	Peptide	Methoxy‐capped diphenylalanine peptide	[6]
Simple	Peptide	Cystamine‐linked diphenylalanine peptides	[7]
Simple	Peptide	Tripeptide spacers and adhesive amino acids	[24]
Simple	Polyoxometalate and polyether	Silicotungstic acid and PEG	[25]
Simple	Polyoxometalate and polyether	Polyethylene glycol and lithium metatungstate	[26]
Complex	Natural and synthetic polyelectrolytes	Sulfonate/sulfate/phosphate/carboxylate‐containing and amine‐based polyelectrolytes	[27]
Complex	Polyelectrolyte and enzyme	Glucose oxidase and DEAE‐dextran	[28]
Complex	RNA and peptide	PolyU RNA and RRASLRRASL peptide sequence	[29]
Complex	Homopeptides and nucleotides	Lys/Arg and Asp/Glu homopeptides or adenosine nucleotides	[30]
Complex	Proteins	Alpha‐synuclein and tau protein	[31]
Complex	Polysaccharides	Chitosan and hyaluronic acid	[32]
Complex	Polysaccharides	Quaternized chitosan and gum arabic	[33]
Complex	Surfactant and Salicylate	Dodecyl trimethyl ammonium bromide and 5‐methyl salicylate	[34]
Complex	Fatty acid	Myristic acid, guanidine hydrochloride	[35]
Complex	Ionic‐liquid based surfactant and salt	3‐methyl‐1‐(octyloxycarbonylmethyl)imidazolium bromide and sodium salicylate	[36]

Physicochemical properties such as molecular weight, charge density, and polarity determine the ability of molecules to undergo LLPS via either simple or complex coacervation. This process is influenced by factors such as concentration, ionic strength, pH value, temperature, solubility, molecular weight, charge density, hydrophobicity, and the presence of electrolytes.^[^ ^22^ ^]^ These variables can be studied individually or in combination. For example, Tang and colleagues showed that elastin‐like peptides and hyaluronic acid form complex coacervates via a temperature‐induced phase transition.^[^ ^23^ ^]^

**Figure 1:**
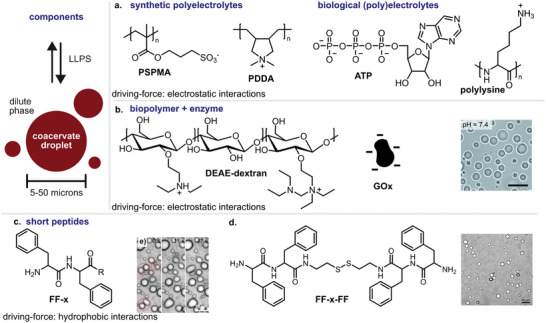
Schematic illustration of coacervate droplet formation and representative building blocks of coacervates. a) Examples of common synthetic polyelectrolytes, synthetic, and biological. b) Coacervates formed by LLPS of glucose oxidase and diethylaminoethyl (DEAE)‐dextran. Scale bar: 10 µm. Adapted from ref. [28] with permission from the Royal Society of Chemistry. c) Diphenylalanine‐based coacervates. Adapted from ref. [6] with permission from Springer Nature. d) Cystamine‐linked phenylalanine dipeptides. Adapted from ref. [7] with permission from Springer Nature.

Synthetic polymers are typically used to form coacervate droplets, and their versatility is an important aspect of their development as a building block for coacervation, as they can be chemically modified to target specific properties, such as charge density.^[^ ^37^ ^]^ For example, Lu and Spruijt systematically studied a large library of oppositely charged macromolecules, including synthetic polyelectrolytes, derivatives of polysaccharides, nucleotides, and oligopeptides, that can self‐assemble into multiphase coacervate droplets by associative phase separation upon mixing with a preferential 1:1 charge ratio.^[^ ^27^ ^]^ The negatively charged polymers ranged from poly(3‐sulfopropyl methacrylate), dextran sulfite, poly(acrylic acid) to adenosine triphosphate, and single‐stranded DNA (**Figure** **1**a). The positively charged polymers included the structurally similar base materials modified with positively charged moieties and the GFP‐K72 protein. The authors demonstrated that combinations of more than one coacervate pair can lead to hierarchical multiphase complex coacervates based on interfacial energies, critical salt concentration, and droplet size.

The charge and solubility of interacting macromolecules are modulated by pH, directly affecting their ability to undergo associative phase separation.^[^ ^38^ ^]^ By fine‐tuning pH, the formation, stability, and morphology of coacervate droplets can be controlled, allowing precise manipulation of these compartments for applications requiring specific environmental conditions, such as enzyme encapsulation and reaction compartmentalization.^[^ ^39^ ^]^

A study by the Martin group demonstrated a hybrid enzyme/polyelectrolyte complex coacervate system (Figure 1b).^[^ ^28^ ^]^ The researchers showed that a cationic polysaccharide‐based diethylaminoethyl dextran (DEAE‐Dex) can form coacervate droplets in the presence of glucose oxidase (GOx) enzyme. The nucleation process occurs at a near 1:1 charge ratio between the polyelectrolyte and the enzyme and at a physiological pH range of 6–8. The catalytic activity of GOx enabled the dynamic assembly of coacervates via enzyme‐driven acidification of the medium in the presence of glucose. The decrease in pH triggered coacervate formation via modulation of the charge ratio between GOx and DEAE‐Dex. This mechanism was used to explore the pH‐modulated formation of multiphase microdroplets for dynamic polyion self‐sorting.

Several groups have focused on developing peptide‐based components for coacervation, moving away from the conventional high molecular weight charged polymers used in complex coacervation.^[^ ^6^, ^7^, ^40^ ^]^ Because of the precise control over molecular structure and design afforded by peptide chemistry, peptides provide a versatile and well‐defined platform for studying the structure–property relationships of coacervates.^[^ ^10^ ^]^ For example, Cao et al. recently developed a short peptide based on a phenylalanine–phenylalanine motif with a modified C‐terminus containing neutral polar groups (Figure 1c).^[^ ^6^ ^]^ The minimalist peptide was able to form coacervate droplets at pH above 7 via simple coacervation. The droplets exhibited a hydrophobic microenvironment suitable for encapsulation of low‐polarity molecules.

Another approach using short peptides was previously presented by the Spruijt group, who developed a peptide derivative consisting of two dipeptides acting as molecular stickers linked by a polar spacer (Figure 1d).^[^ ^7^ ^]^ A cystamine moiety was used to link two phenylalanyl–phenylalanine (FF) dipeptides through their C termini (FFssFF). The peptide produced droplets by simple coacervation, resulting in droplets with properties that depended on the composition of the stickers and spacers. The disulfide bond in the cystamine linker allowed dynamic control of coacervate assembly and disassembly via redox chemistry. In addition to their applications as compartments in catalysis and the design of membraneless organelles, peptide coacervates obtained from short peptides support the hypothesis that primordial biological compartments may have evolved from prebiotic peptides undergoing LLPS modulated by their environment.^[^ ^41^ ^]^

Peptides can form coacervates with proteins, RNA, and other biomacromolecules. For example, the Keating group studied the coacervation properties of peptides and nucleotides.^[^ ^30^ ^]^ Lysine and arginine polypeptides were used as polycations of 1–100 amino acid residues. Nucleotides based on mono‐, di‐, and triphosphates of adenosine (AMP, ADP, and ATP) were used as polyanions. The resulting coacervates showed that shorter polyanions (<30 residues) were better at creating distinct chemical microenvironments and sequestering and preserving RNA duplexes. In particular, the preservation of RNA base pairing in low‐multivalency polyions indicated that the strength of interaction with the negatively charged structure of RNA depends on the number of residues in the polypeptides. These results shed light on the delicate modulation of coacervate–biomolecule interactions relevant to the design of microreactors and cell‐like systems.

Another study by the same group used RNA in combination with peptide to form coacervate droplets as a model for intracellular organelles (Figure 1f).^[^ ^29^ ^]^ Coacervates were obtained from a negatively charged polyuridylic acid RNA (polyU) in combination with a positively charged peptide sequence RRASLRRASL. Control of droplet formation was achieved by changing the phosphorylation state (charge density) of the peptides. The enzyme lambda protein phosphatase (LPP) was used to dephosphorylate the peptides, creating positively charged peptides that subsequently led to coacervation. The process can be reversed by phosphorylating the peptides with protein kinase A. Coacervation was modulated by changing the activity of the kinase and the phosphatase. The polyU/RRASLRRASL system was highly dynamic, responding to the removal/addition of a single phosphate group.

## 3 Dynamic and Controlled Coacervate Formation

In nature, coacervation is a highly dynamic process that leads to the formation of membraneless organelles (MLOs) in cells.^[^ ^42^ ^]^ It occurs spontaneously in the cytoplasm and nucleus to serve several functions, such as storage and protection of cellular material under stress conditions, facilitation of gene expression, and colocalization of biological solutes.^[^ ^43^, ^44^ ^]^ Upon completion of their task, MLOs can undergo disassembly, providing a mechanism for dynamic regulation of cellular processes. This behavior can be mimicked by synthetic coacervates whose assembly/disassembly behavior can be controlled by stimuli such as pH, temperature, redox chemistry, and enzymatic activity.^[^ ^23^, ^45^, ^46^, ^47^ ^]^ Dynamic coacervation is a highly desirable feature for cell‐like microreactors and cell‐like systems in general because it allows adaptive control of chemical processes through selective sequestration of molecules by the coacervate droplets. In addition, dynamic coacervation also allows control over structural properties such as size, morphology, and hierarchical organization, enabling the design of adaptive microreactors and cell‐like systems.

A wide variety of synthetic and biological polymers covalently linked to responsive moieties can exhibit dynamic behavior in the presence of an external stimulus. Examples of responsive moieties and their corresponding stimuli are shown in **Table** **2**.

**Table 2:** Stimulus–responsive coacervate components and their functional groups.

Coacervate components	Responsive moiety	Stimulus	Response	References
Polylysine and ATP	Polylysine side chain	pH	Activation of dormant enzymatic reactions by sequestration	[39]
Charged sodium alginate and cationized silk fibroin	COOH (alginate) and amine groups	pH	Droplet reconfiguration into semipermeable vesicles	[48]
Catalase and DEAE‐Dextran	Amine groups	pH	Droplet size control by active disassembly	[49]
PAA‐g‐PNIPAM and PDMAPAA‐g‐PNIPAM	PNIPAM	Temperature	Transition from flowing to nonflowing hydrogel	[50]
Sulfabetaine methacrylate and 2‐[(methacryloyloxy)ethyl]trimethylammonium	Polymer	Ionic strength	Controlled sequestration of extracellular vesicles	[51]
4,4′‐di(6‐*N*,*N*,*N*‐trimethylammoniumhexyloxy) azobenzene and sodium dodecyl sulfate	Azobenzene groups	Light	Vesicle to droplet transition	[52]
succinylated amylose and trans‐azobenzenetrimethylammonium bromide	Azobenzene groups	Light	Dynamic regulation of enzymatic reaction rate	[53]
K_72_ and ATP	Phosphate groups	ATP generation	Fuel driven droplet growth	[54]
Self‐assembling dipeptide	Phenylalanine groups	In situ coupling	Thermally induced structural transition (sponge‐like to homogenous)	[55]
Arginine‐rich peptide and ssRNA	Arginine and Uracil rings	Divalent cation Mg^2+^	Regulation of droplet chemical and physical properties	[56]
Diallyldimethylammonium chloride (PDDA) and ATP	Phosphate groups	Electric field	Droplet position control	[57]
Fe(CN)_6_^4−^ and polylysine	Fe(CN)_6_^4−^ / Fe(CN)_6_^3−^	Redox	Formation of redox active droplets	[58]
Methionine‐functionalized poly(S‐alkyl‐rac‐cysteines)	Thioether groups	Redox	Redox switchable droplet formation	^[^ ^59^ ^]^
Ruthenium (II) metallolipid and sodium nitrate	Ru	Redox	Redox mediated expansion, contraction, and dissolution	[60]
DEAE‐Dextran and glutamic acid functionalized azobenzene	Azobenzene, amine, and carboxylic acid	Light, pH	Dual responsive droplet formation and dissolution	[61]

The systems listed in Table 2 offer tools for the design of cell‐like microreactors with precise control of reaction environments similar to cellular regulation. One example is the regulation of enzymatic reactions by activating dormant enzymatic reactions through sequestration and colocalization.^[^ ^39^ ^]^ The coacervate systems listed in Table 2 respond to various stimuli including pH, temperature, ionic strength, light, ATP, ions, electric fields, and redox conditions. Each of these stimuli has distinct advantages and limitations. For example, pH‐ and temperature‐sensitive systems allow controlled coacervation, but are less selective and can alter the performance of other components and processes, such as reaction kinetics and catalyst activity and selectivity. Light‐sensitive systems allow reversible transitions but require constant illumination to maintain the system in a constant phase state. Especially in their applications as microreactors, it is important to select stimuli that do not significantly interfere with the intended reactions. For example, light has been shown to promote coacervation and does not interfere with ATP‐independent enzymatic reactions. Therefore, the functionality of each system must be tailored to the specific application.

**Figure 2:**
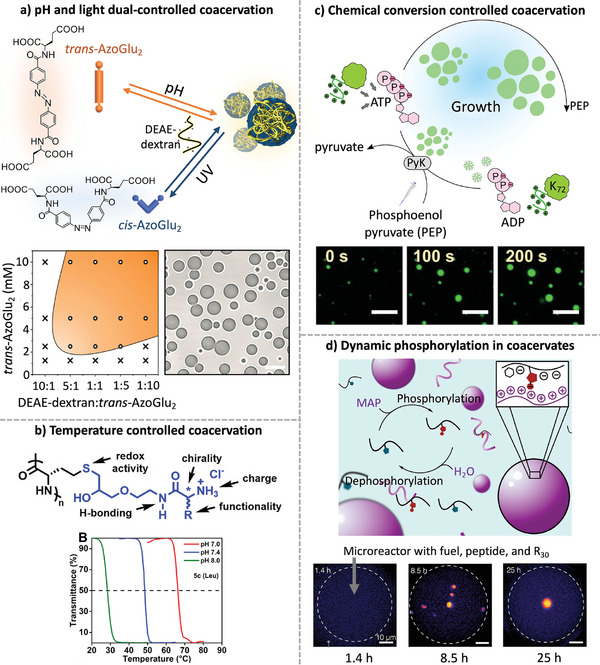
Dynamic formation of coacervate droplets as a consequence of external and internal stimuli. a) Coacervation controlled by pH and light stimuli; scale bar, 10 µm. Adapted from ref. [61] with permission from American Association for the Advancement of Science, Copyright 2021. b) Coacervation controlled by temperature, pH, and ionic strength stimuli. Adapted from ref. [62] with permission from American Chemical Society, Copyright 2021. c) Coacervation as a result of enzymatic conversion of substrates; scale bars, 10 µm. Adapted from ref. [54] with permission from Springer Nature, Copyright 2021. d) Dynamic phosphorylation of peptides within and for coacervate formation; scale bars, 10 µm. Adapted from ref. [63] with permission from Springer Nature, Copyright 2024.

Due to the noncovalent nature of LLPS, the stability and properties of the coacervate phase are highly dependent on the chemical composition of the components and various physical variables. Key factors influencing coacervation include temperature, pH, and ionic strength. More specific stimuli such as light and enzymatic/redox reactions can also be explored by engineering the coacervate composition. For example, the Qiao group developed a coacervate system capable of dynamic self‐assembly in response to two external stimuli, light, and pH (**Figure** **2**a).^[^ ^61^ ^]^

Their design was based on a bolaamphiphilic component with two negatively charged glutamic acids covalently linked to a rigid light‐sensitive azobenzene (AzoGlu2) core. Diethylaminoethyl dextran (DEAE‐Dex) was chosen as the pH‐sensitive cationic polyelectrolyte component. Photoisomerization of AzoGlu2 and protonation of DEAE‐Dex allowed coacervate formation and dissociation to be controlled by UV light and pH, respectively. The dual control allowed the researchers to use these coacervates as reactive membrane organelles capable of processing and computing chemical signals within a proteinosome‐based protocell.

The results demonstrated the potential of responsive coacervates to be used in the development of hierarchical cell‐like materials for chemical computation. Another example comes from the Deming group who developed poly(S‐alkyl‐l‐homocysteine) functionalized with amino acid side chains. (Figure 2b).^[^ ^62^ ^]^ This polypeptide dynamically forms coacervates in response to redox changes, pH value, ionic strength, and temperature within a physiological range. The polypeptides with alanine, valine, leucine, and phenylalanine side chains exhibited different hydrophobicities. The least hydrophobic Ala‐functionalized polypeptide remained soluble up to 90 °C, while the more hydrophobic Val‐, Leu‐, and Phe‐functionalized polypeptides phase separated in a pH‐ and temperature‐dependent manner. Cloud point temperatures decreased with increasing hydrophobicity. This modular design allows fine‐tuning of coacervate properties useful for applications such as drug delivery, tissue engineering, and biosensors.

Coacervate assembly can also be regulated by internal stimuli, such as the product of in situ chemical reactions, which can modulate the behavior and properties of the resulting coacervate droplets.^[^ ^54^, ^63^ ^]^ For instance, the Spruijt group developed active coacervate droplets that can grow while resisting Ostwald ripening. The system involved the formation of complexes of ATP and the protein K_72_, which was controlled by the enzymatic conversion of ADP to ATP (Figure 2c).^[^ ^54^ ^]^ The continuous supply of ADP leads to a local increase in ATP, which causes the recruitment of more protein K_72_, leading to droplet growth. This continuous cycle of conversion of a substrate to a product capable of coacervation provides a platform for chemically regulated compartmentalization and droplet growth. The work also provides insights into the chemical principles that might have driven the early stages of cellular life.

Another example of active droplet formation comes from the Boekhoven group, where dynamic peptide phosphorylation was used to control coacervate assembly and disassembly (Figure 2d).^[^ ^63^ ^]^ The researchers explored the transient phosphorylation of histidine‐containing peptides to control coacervate formation. Potassium monoamidophosphate was used as the phosphorylating agent due to its efficacy with imidazoles. The properties of the droplets depended on the concentration of phosphorylated peptides, which was regulated by a competition between fuel‐driven phosphorylation and hydrolysis of the labile phosphorylated species. This work illustrates an enzyme‐free chemical reaction cycle as a mechanism to control the dynamic formation of coacervate droplets.

The Boekhoven group also developed active RNA/peptide coacervate droplets controlled by a fuel‐driven reaction cycle.^[^ ^64^ ^]^ The reversible assembly of these droplets was controlled by a peptide whose association strength with the RNA component is regulated by its charge state. Droplet formation was favored when a negatively charged aspartate residue on the peptide was converted to its corresponding anhydride in the presence of 1‐ethyl‐3‐(3‐dimethylaminopropyl)carbodiimide (EDC). The anhydride product eventually hydrolyzed back to the precursor peptide, resulting in a transient anhydride state driven by the consumption of EDC. Consequently, the coacervate droplets were able to form in the presence of fuel and disintegrate in its absence. Active coacervate droplets exhibit the characteristics of membraneless organelles, including formation, decay, rapid molecular exchange, and modulation of functionality, making them potential models for the development of cell‐like systems.

## 4 Cell‐Like Microreactors

Biomolecular coacervates can regulate biochemical processes by sequestering proteins and nucleic acids in microenvironments with compositions different from the cytosol.^[^ ^65^ ^]^ Synthetic coacervates can mimic the regulatory properties of these condensed phases in chemical processes. By colocalizing molecules such as reagents and catalysts, they can act as cell‐like microreactors, enhancing chemical reactions or processes by selectively concentrating molecules and modulating reactivity based on changes in the microenvironment.^[^ ^6^, ^66^ ^]^

Recent studies have shown that the affinity of the coacervate phase for different cargoes can be modulated by rational design of the droplet microenvironment using components with different polarities, charge densities, and molecular structures.^[^ ^6^, ^30^, ^67^ ^]^ This tunability of the local droplet microenvironment has opened new avenues for microreactor design, allowing fine control of reactivity and selectivity. Examples of coacervate‐based microreactors are shown in **Table** **3**.

**Table 3:** Coacervate components and their ability to host and promote chemical reactions.

Coacervate components	Reaction components	Chemical reaction	References
Cationic diphenylalanine peptide	Acrylic monomer and photoinitiator	Radical polymerization	[68]
DNA‐pEGA copolymer	Protected dansylfuran, DMAP	Retro‐Diels–Alder	[69]
Oligonucleotides and cationic azobenzene	End‐phosphorylated oligonucleotides, EDC	Ligation of oligonucleotides	[70]
ATP and DYFR_9_ peptide	Tyrosine and tyrosynase	Enzymatic synthesis of oxidized phenols	[71]
PAA, *N*,*N*′‐dimethylethylenediamine	Glucose oxidase, glucose	Enzymatic oxidation	[72]
Carbon dots and PDADMAC	CDs, nitroarenes, ferrocyanide	Hydrogeneration and photoreduction	[73]
DEAE and carboxymethyl dextran	de novo enzyme, ortho‐phenylenediamine	Enzymatic oxidation	[74]

**Figure 3:**
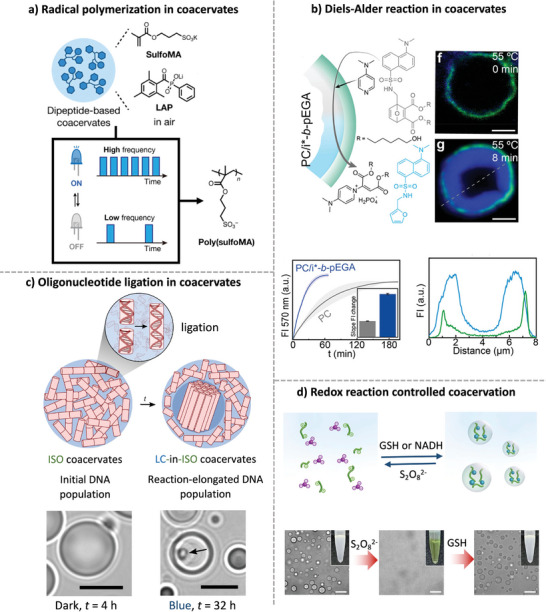
Coacervates as compartments to facilitate chemistry. a) Radical polymerization process within dipeptide coacervates. Adapted from ref. [68] with permission from American Chemical Society, Copyright 2022. b) Diels–Alder reaction in DNA‐based coacervates with tunable microenvironment; scale bars, 2 µm. Adapted from ref. [69] with permission from the authors, Copyright 2023. c) Oligonucleotide ligation in coacervates for inducing phase transition and morphology change; scale bars, 5 µm. Adapted from ref. [70] with permission from Springer Nature, Copyright 2023. d) Redox controlled coacervation; scale bars, 10 µm. Adapted from ref. [58] with permission from Springer Nature, Copyright 2023.

The Hamachi group used dipeptide coacervates as microreactors for radical polymerization. They designed a dipeptide derivative containing a diphenylalanine tert‐butyl motif that produced coacervate droplets (**Figure** **3**a).^[^ ^68^ ^]^

The researchers took advantage of the unique microenvironment of these coacervates, which can sequester a photoinitiator and methacrylate monomers, to perform light‐triggered radical polymerization within the droplets. The hydrophobic microenvironment within the coacervates increased the concentration of reactants and photocatalyst and allowed the inhibitory effect of molecular oxygen on polymerization to be regulated by the light pulse frequency. The resulting polymer formation resulted in changes in coacervate morphology and viscosity due to competition between reactive radicals from the methacrylate monomer and molecular oxygen.

The stimuli‐responsiveness of coacervate components offers a dynamic way to control the performance and functionality of cell‐like microreactors. For example, the Walther group developed a DNA‐based coacervate using a temperature‐switchable DNA‐*b*‐polyethylene glycol‐acrylate block copolymer that undergoes phase segregation at elevated temperatures (Figure 3b).^[^ ^69^ ^]^ Upon heating, the coacervate droplet undergoes thermoreversible phase segregation, forming a distinct hydrophobic compartment within the droplet. This hydrophobic compartment enhanced the solubility of hydrophobic molecules and increased the reactivity of bimolecular reactions, as demonstrated in a retro‐Diels–Alder reaction involving a protected dansylfuran and 4‐dimethylaminopyridine. The reaction produced a hydrophobic dansylfuran fluorophore that preferred the hydrophobic microenvironment and is readily detectable. This work demonstrates the versatility of coacervates in creating programmable cell‐like microreactors for applications in synthetic chemistry and the development of synthetic cell models.

Because of the liquid‐like properties of coacervates and their responsiveness to a variety of stimuli, it is possible to control phase transition, droplet morphology and the properties of their microenvironment.^[^ ^75^ ^]^ The Martin group developed coacervate droplets that promoted nonenzymatic oligonucleotide polymerization followed by phase transitions and the formation of multiphase droplets (Figure 3c).^[^ ^70^ ^]^ They exploited the light‐mediated *trans–cis* properties of azobenzene to dynamically modulate coacervate assembly and dissolution in the presence of end‐stacking oligonucleotides. Different phases formed as a function of ionic strength and light conditions, with ligation reactions being most efficient in liquid crystalline coacervates due to their fluid and ordered environment. This demonstrates that coacervate droplets can significantly enhance nonenzymatic oligonucleotide ligation, which has implications for the design of microreactors and cell‐like systems, as well as providing insights into the development of protocells and the origin of life.

Another interesting example from the Spruijt group showed that prebiotically relevant ferricyanide can be used to created redox‐active coacervates (Figure 3d).^[^ ^58^ ^]^ Mixtures of Fe(CN)_6_^4−^ or Fe(CN)_6_^3−^ with cationic polypeptides formed coacervates. The stability of the droplets depended on the oxidation state of the anion, and could be disintegrated by addition of S_2_O_8_^2−^ to cause the oxidation of Fe(CN)_6_^4−^ to Fe(CN)_6_^3−^. In addition to reversible formation, droplets containing ferricyanide were redox active and enabled the formation of amide bonds between amino acids and alpha‐amidothioacids within the droplets. This study demonstrated the potential of active redox coacervates as versatile protocell models capable of dynamically localizing and enhancing prebiotically relevant chemical reactions.

The first types of cell‐like systems typically consisted of an internal dilute aqueous medium contained by capsules or vesicles encapsulating active species, such as biocatalysts, or other compartments.^[^ ^76^, ^77^, ^78^, ^79^, ^80^ ^]^ Recently, coacervates have been recognized as a versatile compartment for the construction of cell mimicking systems.^[^ ^81^, ^82^, ^83^ ^]^ Properties such as high adaptability, molecular crowding, and tunable microenvironments make them excellent compartments for the development of cell‐like systems with life‐like properties. Examples of the components and structures of coacervate‐based cell mimicking systems are listed in **Table** **4**.

**Table 4:** Coacervate components and their role in multicompartmentalized cell‐like systems.

Coacervate components	Structure	Role in the organization	References
Polydiallyldimethylammonium chloride and DNA	Membranized coacervate droplet	Membrane permeability regulation, reversible multicompartmentalization	[84]
Quaternized amylose (Q‐am), Carboxymethylated amylose (CM‐am), and NTA‐amylose	Membranized coacervate droplet	Controlled loading of biomolecules	[82]
ATP and poly(allyl amine hydrochloride) (PAH)	Coacervate droplets in vesicles	Signal‐driven multicompartmentalization	[85]
CM‐am, Q‐am, and block copolymer PEG–PCLgTMC	Membranized coacervate droplet	Cytosol‐like matrix containing polymersome‐based artificial organelles	[86]
Arginine‐glutamate‐arginine tripeptide, polyphosphate, and Mn^2+^	Coacervate‐in‐coacervate	Multicomparmentalized droplet for sequestration and protection of biomolecules	[87]
CM‐dextran, DEAE‐dextran, PDDA, and DNA	Coacervate‐in‐coacervate	Organization of enzymatic reactions	[88]
Guanidinium and myristic acid	Proteinosome‐in‐coacervate	Host–guest protocell construct	[89]
CM‐Dex with PDDA	Artificial organelles‐in‐coacervate	Multicompartmentalized coacervate protocells	[83]

Liposomes and polymersomes are commonly used in the construction of artificial cells because their structure closely mimics some properties of natural cell membranes, such as a flexible bilayer structure, amphiphilicity and selective permeability.^[^ ^90^, ^91^ ^]^ Coacervates, which function as membraneless compartments, can be incorporated into liposomes, polymersomes, or proteinosomes to create multicompartment hybrid cell‐like systems. There are two ways to induce coacervation in giant vesicles. One is based on entrapment of preformed coacervates in vesicles or encapsulation of coacervate materials and use of stimuli such as pH and light to induce coacervation. The other is based on encapsulating only one of the materials inside the vesicle, while the other is freely distributed in the outer medium and subsequently permeates the membrane, either by increasing the permeability of the membrane layer through functional modules or protein channels, or by creating a compartment with intrinsic permeability for macromolecules such as proteinosomes.^[^ ^17^, ^39^, ^92^ ^]^

For example, Love and colleagues demonstrated the reversible formation of coacervate‐based subcompartments within lipid vesicles.^[^ ^39^ ^]^ The subcompartment was a single coacervate droplet composed of polylysine (PLys) and adenosine triphosphate (ATP) that could dynamically self‐assemble or disassemble in response to pH changes. The lipid membrane allowed diffusion of ions from the external medium, enabling the internal coacervation process. This coacervate‐in‐liposome cell‐like system serves as a model for the dynamic colocalization of molecules. The researchers demonstrated that the coacervate increased the local concentration of an enzyme (formate dehydrogenase) and its substrates, creating favorable conditions for the activation of the enzymatic reaction.

In another study by the Dekker group, PLys and ATP were used as components for coacervation within liposomes, but one of the components (PLys) was encapsulated while the other (ATP) could only access the interior of the droplet through a bilayer‐spanning protein pore.^[^ ^92^ ^]^ The spatiotemporal control achieved by this approach illustrates the versatility of the hybrid vesicle–coacervate system to mimic the dynamic compartmentalization and hierarchical architecture of natural cells.

The permeability of the outer membrane in vesicle–coacervate cell mimics can be controlled by adjusting the composition of the vesicle membrane. The Lee group demonstrated this by using mixtures of polybutadiene‐*b*‐poly(ethylene oxide) and poly(ethylene glycol)‐*b*‐poly(propylene glycol)‐*b*‐poly(ethylene glycol) to form synthetic polymeric cells surrounded by a semipermeable membrane.^[^ ^85^ ^]^ The signal‐driven formation of internal PAH/ATP coacervates was achieved by membrane‐mediated diffusion of phosphoenolpyruvate, which led to an increase in the internal concentration of ATP within the vesicles through the action of pyruvate kinase.

**Figure 4:**
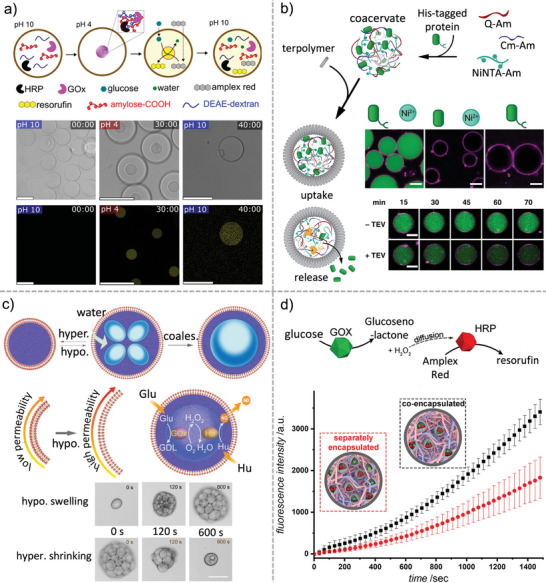
Multicompartmentalized cell‐like systems. a) The formation of pH‐responsive carboxyamylose and diethylaminoethyl‐dextran coacervates in polymersomes as subcompartments for sequestering a cascade reaction to produce resorufin; scale bar, 50 µm. Adapted from ref. [93] with permission from the Authors, Copyright 2023. b) Schematic illustration of the uptake and release mechanism of His‐tag proteins into polymer‐stabilized coacervates, confocal micrographs of the uptake of sfGFP protein with and without a His‐tag or Ni^2+^ (upper), and the release of sfGFP upon exposure to TEV protease (lower); scale bars, 10 µm. Adapted from ref. [82] with permission from Springer Nature, Copyright 2020. c) Schematic illustration of the swelling and shrinking mechanism of coacervates upon exposure to hypotonic or hypertonic environments, leading to the change in membrane permeability (left) and micrographs of PDDA/DNA coacervates droplets during hypotonic swelling and hypertonic shrinking (right); scale bar, 15 µm. Adapted from ref. [84] with permission from American Chemical Society, Copyright 2023. d) Reaction kinetics of the GOx/HRP cascade reaction determined with fluorescence intensity of resorufin for protocells having coacervate and polymeric organelles. Reproduced from ref. [86] with permission from American Chemical Society, Copyright 2019.

This work demonstrates a sophisticated two‐step control of internal coacervate formation that requires proper signal diffusion across a selective membrane and signal processing by an enzyme. Another example of the effect of membrane composition on the dynamic formation of internal coacervates was presented by Ivanov and colleagues.^[^ ^93^ ^]^The researchers used a specific combination of polybutadiene‐*b*‐poly(ethylene oxide) and oleyl alcohol to form polymersomes permeable to protons and molecules with molecular weights up to 0.5 kDa. Coacervation of carboxyamylose and diethylaminoethyl‐dextran in response to a reduction in pH was used to create dynamic subcompartments for sequestering an enzymatic cascade of reactions to produce resorufin (**Figure** **4**a). This approach provides a platform for the rapid and efficient construction of robust, adaptive microreactors that can be used in catalysis, biosensing, and cell mimicry.

The molecularly crowded environment of coacervates more closely resembles the cell cytoplasm in terms of molecular crowding than the dilute interior of membrane‐based vesicles. It is important, however, to note that the cytoplasm is much more complex, containing highly organized network of biomolecules, organelles, and dynamic structures that interact in intricate ways. Although coacervates are ideal for cargo loading, the partitioning of components into coacervates depends on their charge and hydrophobicity.

The van Hest group reported an elegant system for programming the sequestration and release of different macromolecules from coacervates (Figure 4b).^[^ ^82^ ^]^ The protocells were prepared using coacervates derived from quaternized amylose (Q‐Am), carboxymethylated amylose (Cm‐Am), and amylose modified with a nitrilotriacetic acid group (NTA‐Am). These coacervates were stabilized by a terpolymer membrane. When coacervation occurred in a buffer containing Ni^2+^, polyhistidine‐tagged (His‐tagged) proteins were sequestered in the coacervates because the His‐tag binds strongly to the Ni^2+^–NTA complex. A cargo release mechanism was also presented in which proteins were released from the coacervates upon addition of a protease that specifically cleaves the His‐tag from the proteins.

The combination of adaptable coacervate compartments and semipermeable membranes can produce cell‐like systems and microreactors capable of dynamic structural reorganization. The Liu group developed a phospholipid‐stabilized coacervate system as a model protocell capable of osmotic‐induced reconfiguration (Figure 4c).^[^ ^84^ ^]^ The coacervates, consisting of polydiallyldimethylammonium chloride (PDDA) and DNA, exhibited reversible volume changes in response to hypotonic and hypertonic environments. Hypotonic swelling produced multichambered coacervate vesicles with increased volume and increased membrane permeability, which facilitated and regulated enzymatic reactions within the microreactors. The increased membrane permeability under osmotically induced tension enhanced mass transfer, allowing the initiation and enhancement of protease‐based hydrolysis and glucose oxidase/hemoglobin‐mediated nitric oxide (NO) production within the swollen coacervate vesicles. This system provides a mechanism for enhancing the in vitro vasodilator response in thoracic artery rings, suggesting that changes in osmolarity can be coupled to microreactor–living cell interactions.

A compartment‐in‐compartment architecture enables the development of cell‐like systems with internal active compartments that mimic the spatial and functional organization of eukaryotic cells. An example of a cell‐like microreactor with synthetic organelles incorporated into membranized coacervate droplets was reported by Mason and colleagues (Figure 4d).^[^ ^86^ ^]^ The protocells were generated by spontaneous sequestration of polymersomes loaded with enzymatic cargo in amylose‐based coacervates, which were then surrounded by a polymeric membrane. The polymersomes were prepared from poly(ethylene glycol)‐*b*‐poly(caprolactone‐gradient‐trimethylene carbonate) (PEG‐PCLgTMC) block copolymers. Coencapsulation of enzymes in these multicompartment microreactors resulted in an increased overall reaction rate. This was demonstrated using an enzymatic reaction involving glucose oxidase and horseradish peroxidase. In addition, the authors showed that encapsulation can be used to separate incompatible enzymes, illustrating how compartmentalization can enable the design of multicatalyst systems.

## 5 Conclusion and Outlook

Synthetic coacervates serve as versatile analogues of biomolecular condensates, providing a platform for the systematic study of LLPS and enabling the design of biomimetic materials and systems with life‐like properties. As discussed in this review, a wide range of materials are capable of undergoing LLPS, including synthetic polymers, biopolymers, and small (bio)molecules, providing a versatile toolbox for designing adaptive soft compartments with unique tailored microenvironments. The modularity of coacervates has been demonstrated through techniques enabling droplet membranization and methods for constructing hierarchical multicompartment architectures. These advances pave the way for the development of sophisticated adaptive cell‐like microreactors, that mimic the structure and behavior of natural systems.

Beyond simple concentration effects, coacervates have demonstrated properties that bring life‐like behavior to synthetic chemistry. Coacervates provide a platform where different types of catalysts and subcompartments can be combined to create complex chemical systems. In addition, the properties of the condensed phase can be modulated by external conditions or chemical signals, allowing control over the properties and performance of chemical reactions occurring within the droplets. Other properties such as selective partitioning of molecules, morphology, and rheological properties can also be explored to control chemical processes beyond the capabilities of other types of compartments such as vesicles, micelles, and capsules. In contrast to biological condensates, synthetic coacervates are simpler, although our understanding of their sequestration capabilities is still evolving. Studies of coacervates with simple building blocks, such as short peptides, have the potential to uncover encapsulation principles based on quantifiable hydrophobicity, hydrogen bonding, and electrostatics.

Researchers are still uncovering the driving forces behind LLPS. Details of the mechanisms that lead to enhancement and selectivity of chemical reactions in coacervate‐based microreactors are still not fully understood.^[^ ^94^ ^]^ While computer simulations and synthetic chemistry, especially polymer and peptide chemistry, have provided invaluable information about the behavior of small and large molecules during LLPS, the development of general rules remains challenging due to the variety of molecular interactions and structural properties of the components.^[^ ^4^ ^]^ Recent efforts to create minimalist coacervates, composed of the smallest possible components, are helping to simplify the understanding of structure–property relationships in coacervate droplets and guide the development of droplets with highly tailored properties. Analytical tools and methods to characterize coacervates are also in need of development. Due to their typically large size (1–100 microns), properties such as surface charge are difficult to accurately determine using light scattering methods. Additionally, coacervate droplets are typically characterized by microscopy, which limits the acquisition of large data sets. Therefore, new techniques are needed and being developed to characterize properties such as size distribution, interfacial composition, rheology, and mechanical properties of these fascinating and versatile materials. Recent developments in this area, such as microelectrophoresis and microrheology are promising and in high demand.^[^ ^95^, ^96^ ^]^ Developments in droplet microfluidics are showing promising results in this area, particularly in the manipulation of large numbers of droplets under controlled conditions and rapid data acquisition.^[^ ^97^ ^]^

A major design challenge for coacervates is their intrinsically low stability, which requires membranization, crosslinking or controlled conditions to prevent droplet dissolution, coalescence and surface wetting.^[^ ^14^, ^19^, ^20^, ^98^ ^]^ The sensitivity of the phase behavior of coacervates in the presence of other components also complicates the practical application of coacervates in microreactors, especially in multicomponent mixtures involving reactants, products, and catalysts. The sensitivity of coacervates to dilution, pH and temperature changes, and ionic strength is amplified in applications where coacervates are exposed to biological fluids and in biological applications, such as drug delivery.

Coacervates are at the interface of cell biology and materials science, and advancing the field requires a combination of techniques and expertise from multiple disciplines, including chemistry, biophysics, cell biology, and computer simulation. An interdisciplinary approach is essential to realize the full potential of coacervate‐based systems. Continued exploration of coacervates as adaptive, life‐like compartments will unlock their full potential and lead to significant advances in both fundamental understanding and practical applications, including drug delivery, biomimetic chemistry, synthetic biology, (bio)molecular sensors, biomaterials, and more.

## Conflict of Interest

The authors declare no conflict of interest.
